# Novel insights into RNAi off-target effects using *C. elegans *paralogs

**DOI:** 10.1186/1471-2164-8-106

**Published:** 2007-04-19

**Authors:** Jean-François Rual, Niels Klitgord, Guillaume Achaz

**Affiliations:** 1 Center for Cancer Systems Biology (CCSB) and Department of Cancer Biology, Dana-Farber Cancer Institute and Department of Genetics, Harvard Medical School, Boston, Massachusetts 02115, USA; 2 UMR7138 & Atelier de Bioinformatique, Université Pierre & Marie Curie, Paris, France

## Abstract

**Background:**

In the few years since its discovery, RNAi has turned into a very powerful tool for the study of gene function by allowing post-transcriptional gene silencing. The RNAi mechanism, which is based on the introduction of a double-stranded RNA (dsRNA) trigger whose sequence is similar to that of the targeted messenger RNA (mRNA), is subject to off-target cross-reaction.

**Results:**

We use a novel strategy based on phenotypic analysis of paralogs and predict that, in *Caenorhabditis elegans*, off-target effects occur when an mRNA sequence shares more than 95% identity over 40 nucleotides with the dsRNA. Interestingly, our results suggest that the minimum length necessary of a high-similarity stretch between a dsRNA and its target in order to observe an efficient RNAi effect varies from 30 to 50 nucleotides rather than 22 nucleotides, which is the length of siRNAs in *C. elegans*.

**Conclusion:**

Our predictive methods would improve the design of dsRNA and ultimately the use of RNAi as a therapeutic tool upon experimental verification.

## Background

The discovery of RNA interference (RNAi) has been an important breakthrough in biology in the past years [1,2]. Although RNAi-related phenomena have been described for decades [3], it is only recently that the silencing mechanism has been understood at the molecular level [4]. In *Caenorhabditis elegans*, Fire and colleagues described RNAi as a sequence-specific gene-silencing event based on the introduction of a double-stranded RNA (dsRNA) template having sequence similarity with the targeted messenger RNA [5]. Since then, the RNAi pathway has been shown to be conserved in a wide variety of eukaryotic organisms [6].

The physiological functions of RNAi range from immune response by degradation of exogenous genetic material [7] to development by regulation of gene expression [8-10]. In recent years, RNAi has been extensively used as an experimental tool for the analysis of gene function [11,12]. As our understanding of the underlying molecular machinery improves, the potential use of RNAi for therapy is becoming more evident [13,14].

In the RNAi pathway, long dsRNAs or short hairpin RNAs are processed and digested into small interfering RNAs (siRNA; 21 to 23 nucleotides) by Dicer, an RNase III family member [10,15]. After incorporation of the siRNA in the RNA-induced silencing complex, the siRNA-protein complex degrades mRNAs having sequence complementarity to the siRNA [4]. Interestingly, the RNAi machinery has also been shown to mediate translational repression or induce chromatin modification [16].

Since interference is based on sequence recognition, targeting a gene by RNAi can give rise to the silencing of another gene with similar sequence [17]. This phenomenon is referred to as off-target effect or cross-reaction and can occur through mRNA degradation or through translational repression [4]. Although RNAi phenotypes are generally assumed to be due to the sole knock-down of the targeted gene, they can also be due to multi-gene silencing. Numerous genome-wide RNAi analyses have been performed, *e.g.*, for *C. elegans *[11] and *Drosophila melanogaster *[12]. Due to the issue of cross-reaction, RNAi data should be filtered prior to their analysis. In addition, accurate knowledge of RNAi specificity is critical when considering the use of RNAi as a therapeutic technology [13,14]. In this context, it is crucial to know how much similarity is necessary to observe RNAi off-target cross-reaction.

To assess identity requirements for RNA interference in *C. elegans*, Parrish *et al*. [18] used a series of altered GFP coding regions with different degrees of similarity to the transgene target. They found effective interference with dsRNAs (~520 nucleotides long) that were 96% identical to the target sequence (193 nucleotides maximum uninterrupted identity), less effective interference with a trigger that was 88% identical (41 nucleotides maximum uninterrupted identity), and no interference with dsRNA triggers that were 78% identical to the target (23 nucleotides maximum uninterrupted identity). Different studies based on mRNA expression profile analysis led to contradictory conclusions regarding RNAi target specificity [19-21]. For *C. elegans *RNAi data, Fraser *et al*. [22] used 200 nt with 80% identity as a threshold. Interestingly, *in silico *examination of potential cross-reaction as a function of siRNA length led to the conclusion that target specificity and low probability of off-target effects were optimally balanced for siRNAs of 21 nucleotides [23]. Finally, for data repositories such as RNAiDB [24], tools have been developed for analysis and visualization of the RNAi data as well as their potential risk of "contamination". Although these experiments give some indication of the sequence identity requirement, the question of how much similarity over how much length is necessary to observe off-target cross-reaction remains open.

## Results and discussion

Using the RNAi phenotypes of paralogous genes in *C. elegans*, we devised a strategy to estimate the minimum degree of similarity needed to observe off-target effects. Hereafter, any gene that exhibits any kind of RNAi phenotype will be further referred to as a "PH gene" without further consideration of the phenotypic details. Similarly, any gene that was targeted by one or more RNAi experiments and for which no phenotype is described will be defined as a "WT gene". We extracted all 540 pairs of strict duplicates (gene families with only two members) for which both copies were targeted by RNAi (see Additional file 1). These pairs can be sorted according to the phenotypic class of both members: 393 (0.73) "WT/WT" pairs consist of two WT genes, 38 (0.07) "PH/PH" pairs of two PH genes and the remaining 109 (0.20) "WT/PH" pairs have one WT member and one PH member.

In order to predict whether these duplicates exhibit off-target cross-reaction, we elaborated a model where all duplicates are subject to off-target cross-reaction. In this model:

a) The probability of occurrence of PH/PH duplicate pairs is the same as the probability of occurrence of PH genes within singletons (*i.e.*, genes with no paralogs). The probability of a gene to be duplicated and further preserved being only very weakly correlated with the phenotypic class a gene (Rual and Achaz, unpublished data), therefore, if at least one copy of the pair inherits the phenotypic class of their ancestral gene, we expect this first assumption to be true.

b) Some PH genes are annotated as WT genes due to the important fraction of false negatives. Actually, the fraction of false negative in large-scale RNAi experiments can be as high as 0.5 but the fraction of false positive is extremely low. In our dataset, we estimate the fraction of false negatives to be 0.37 (Methods).

c) Both genes in a duplicate pair give rise to the same RNAi phenotype. This last assumption seems reasonable when off-target cross-reaction happens, since the targeting of one copy would also knock down the other copy.

Under such a model, we expect 0.12 of PH/PH pairs, 0.14 of WT/PH pairs and of 0.74 WT/WT pairs. We then computed the probability (using a multinomial cumulative likelihood framework) of the entire set of 540 duplicate pairs to fit our model. We observed that the fractions of WT/WT, WT/PH and PH/PH of the entire set of pairs do not fit our model (N = 540; observed frequencies: 0.73 WT/WT, 0.20 WT/PH and 0.07 PH/PH; expected frequencies: see above; *P *< 10^-7^). From this result, we concluded that not all duplicates exhibit off-target effects. This result is expected because only very similar duplicates should exhibit off-target cross-reaction.

**Figure 1:**
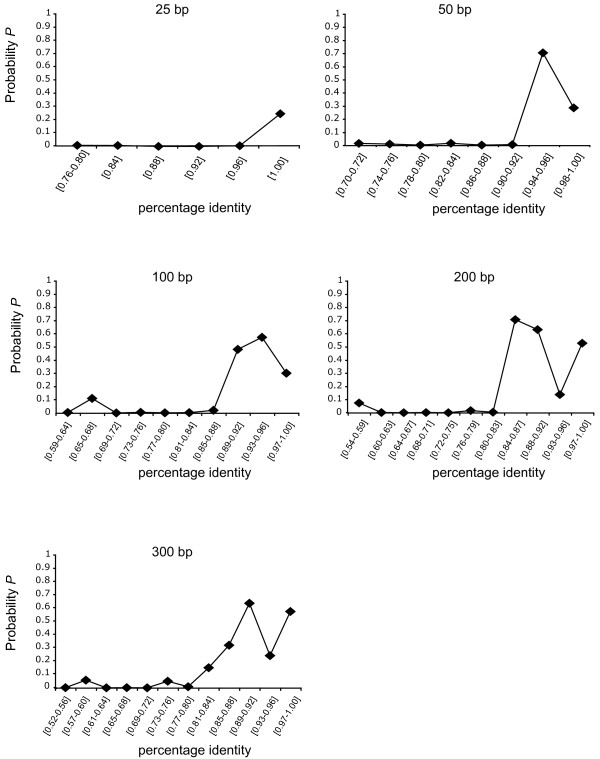
**Off-target effect predicted to occur with genes having high degree of sequence similarity with the RNAi clones**. In this figure, we represent the probability that duplicate pairs can be subject to off-target cross-reaction as a function of the identity between their coding sequences and the sequences of each other's RNAi clones. The maximum percentage identity between the genes and the RNAi clones was calculated over various lengths: 25 nt, 50 nt, 100 nt, 200 nt and 300 nt. The probability is the likelihood that the data (number of WT/WT, WT/PH and PH/PH pairs) fit a model where all duplicates are subject to off-target cross-reaction (see text). From this, we predict that only pairs having high degree of sequence similarity with the RNAi clones (100% over 25 nt, ≥94% over 50 nt, ≥89% over 100 nt, ≥84% over 200 nt and ≥81% over 300 nt) exhibit off-target cross-reaction.

Accordingly, we sorted the pairs using the degree of sequence similarity between the genes and the corresponding RNAi clones (see Methods for details). Since we did not want to assume a sequence length for off-target cross-reaction, we chose to estimate the percentage identity over several lengths, *i.e.*, over 25, 50, 100, 200 or 300 nucleotides. Sixteen out of the 540 pairs had at least one of their genes longer than 5 kb and identity were not estimated because of memory consumption. For each selected length, we grouped the pairs according to their percentage identity. For each group, we counted the number of WT/WT, WT/PH and PH/PH pairs and estimated the corresponding likelihood of these observed counts to fit the model of complete off-target cross-reaction. The higher the likelihood, the higher the chance this group is sensitive to off-target effects. Results (Figure 1) show that only genes having high degree of sequence similarity with the RNAi clones, *i.e.*, 100% over 25 nt, ≥94% over 50 nt, ≥89% over 100 nt, ≥84% over 200 nt and ≥81% over 300 nt, fit well with the model (*i.e. P *> 0.2 for high identities). This strongly suggests that off-target effects occur when the percentage identity exceeds these thresholds. As sequence identity decreases, the likelihood of the data to fit the model decreases as well, illustrating the weaker effect of off-target.

To better understand sequence recognition requirements of the RNAi machinery, we looked for the minimum length of high-similarity stretch between the dsRNA and its target necessary to observe efficient RNA interference. More precisely, we calculated the minimal length over which sequence similarity is necessary to have a high likelihood to observe off-target cross-reaction. This is not a trivial problem because pairs that are very similar to the RNAi clone over 25 nt are usually also very similar over 50 nt (and so on). Consequently, to address this question of minimal length, we selected pairs whose maximum percentage identity with the RNAi clones is above the threshold over 25 nt (100% over 25 nt) and, in the same time, below the threshold over 50 nt (<94% over 50 nt). If such pairs exhibit a high likelihood of off-target cross-reaction, it suggests that having 100% identity over 25 nt is sufficient to observe off-target effects. On the other hand, if this likelihood is low, it suggests that having 100% identity over 25 nt is not sufficient to observe off-target effects. Out of 17 such pairs, 14 are WT/WT, 3 are WT/PH and none are PH/PH. The corresponding likelihood is relatively low (*P *= 0.059), suggesting that these pairs may be not subject to off-target cross-reaction and that the minimum length may be more than 25 nt. To compute more precisely the minimum length on which a high identity is necessary to observe off-target cross-reaction, we applied the same strategy to compare 25 nt with 30 nt, 30 nt with 40 nt, etc. Results (Table 1) show that the data fit well the model (*i.e. P *> 0.3) for pairs with high identity on 40 nt only (not on 30 nt or on 50 nt). On the contrary, pairs with high identity on 25 nt only or on 60 nt only fit poorly the model (*i.e. P *~ 0.05). Keeping in mind that our analysis is performed with limited amount of data, it suggests that dsRNA having sequence similarity over 30 to 50 nucleotides (~twice the size of siRNAs) are optimal to observe efficient off-target cross-reaction and, by extrapolation, RNAi in *C. elegans*.

It is important to distinguish between "the minimum length of the dsRNA" and "the minimum length of high-similarity stretch between the dsRNA and its target" necessary to observe an efficient RNAi effect. Our analysis studies the later one. Our results do not imply that, overall, 30–50 nt dsRNAs are the most efficient in an RNAi experiment. On the contrary, in *C. elegans*, long dsRNA molecules tend to be much more efficient than small dsRNA molecules for reasons not only related to the interaction with the target mRNA but also to the initiation of the systemic effect and amplification of the RNAi effect. Here our results suggest that a stretch of 30–50 nt of high similarity inside the dsRNA molecule is sufficient to observe an efficient RNAi. A systematic experimental analysis of the minimum length of the dsRNA molecule having a 30–50 nt stretch of high similarity with targeted gene would complement our study. Moreover, we would like to mention that our results are based on the assumption that only one stretch of high similarity is necessary to observe off-target reaction. Therefore, our method does not assess whether one or multiple stretches of high similarity are necessary.

Our estimated range is in agreement with the current molecular model of the RNAi machinery. Indeed, for each dsRNA molecule having one fragment of 22 nucleotides identity with the targeted mRNA, the chance to have an siRNA of exactly 22 nucleotides identity with the same mRNA after Dicer processing is one out of 22. However, for dsRNA molecule having one fragment of 44 nucleotides identity with the targeted mRNA, the chance to have an siRNA of exactly 22 nucleotides identity with the same mRNA after Dicer processing is 100%.

To date, in mammalian cells, a typical RNAi experiment is performed by using conventional 21-mer synthetic RNA duplexes. In addition to the limitation of using long dsRNA triggers that can induce the non-specific interferon response, the choice of 21-mer long dsRNAs as RNAi triggers was mainly guided by the observation that 21-mer siRNAs are key players in the RNAi machinery (*i.e.*, product of the dsRNA digestion by Dicer and component of the active RISC complex) [4]. However, recent studies demonstrate that, in mammalian cells, synthetic RNA duplexes 25–30 nucleotides in length can be up to 100-fold more potent than corresponding conventional 21-mer siRNAs [25]. The enhanced efficiency is attributed to the fact that longer dsRNAs are substrates of Dicer (while 21-mer siRNAs are only the products of the digestion). Indeed, Dicer would directly link the production of siRNAs to their incorporation in the RISC complex [26]. Likewise, although siRNAs are ~22 nucleotides long in *C. elegans*, our results suggest that sequences with similarity to the target mRNA over a length of 30 to 50 nucleotides are more efficient for RNAi.

We would like to mention that we are aware that the counts we used here can be sometimes low (especially in Table 1). However, the trend seems clear enough that we think our results are meaningful. Corroborating our results with new data sets from other species will allow to explore whether the results we report here are a specificity of *C. elegans *or constitute a more general trend.

Finally, we think that our predictions are ripe for experimental verifications using directed experiments. For example, one can try to induce interference with a non-duplicated target gene with various sequences construction (a single stretch of a chosen length sharing a chosen similarity with the target) and consequently analyze the RNAi phenotype as well as the mRNA expression level, possibly by real-time RT-PCR.

## Conclusion

Our analysis represents a novel approach to estimate the threshold of sequence identity susceptible to off-target cross-reaction in an RNAi assay (≥95% identity over 40 nucleotides in *C. elegans*). While enormous amounts of phenotypic data are being generated for many organisms using RNAi, this strategy allows flagging potential false positive in RNAi datasets. The RNAi data of genes (*i.e.*, all genes, not only duplicates) having greater than 95% identity over 40 nucleotides with another genes should be interpreted cautiously and tagged as potential false positives. We propose that similar strategies and criteria should be applied for cautious interpretation of RNAi data from any organisms. It is noteworthy to mention that our approach may be extended to study additional features having impact on RNAi cross-reaction. Those features include, for example, positional effect of mismatches, effects of UTRs, thermodynamical stability, or prevalence/exclusion of certain nucleotides at different positions. In addition, a better understanding of the off-target effect phenomenon should allow a better application of RNAi as an experimental tool or as a therapeutic approach.

## Methods

### Genome sequence

For *C. elegans *genome sequence and related genome annotation, we used version WS112 available at WormBase [27]. There are 19,920 predicted protein encoding genes in this release. For genes with more than one splicing variant, we arbitrarily chose the 'a' variant.

### Paralogy

To assign paralogy, we pre-selected potential paralogs using BLASTP of all translated coding sequences against themselves. All coding sequences having one or more blast hits (other than themselves) with an e-value smaller than 10^-30 ^(9,166 for *C. elegans*) were retained, translated and were further aligned together with an end-gap-penalty free global alignment [28], using the BLOSUM62 matrix. Each alignment score was then turned into a z-score. This was done by using all other alignment scores involving one of the two genes as a reference distribution. From this distribution, we calculated the mean and the standard error and computed the z-score as (score-mean)/stderr. We then empirically chose a cut-off of z-score >= 10 to assign paralogy between two genes. This leads to 8,087 (41% of all genes) *C. elegans *paralogs (grouped in 1,547 families). Here, we decided to keep only the 675 families of two members to keep the situation as simple as possible. The complicated relationships that can happen in a three or more partners cross-reaction can then be ignored.

### RNAi phenotypes

The *C. elegans *RNAi data [11,22,29-31] were retrieved from WormBase WS112 [27]. In addition, we used data available from [32]. All in all, out of the 19,920 predicted genes in *C. elegans*, 17,270 (86.7%) have been screened at least once in an RNAi assay out of which 2,543 (15%) gave rise to a detectable phenotype different from WT in at least one RNAi screen. Gene duplicates represent 42% (7,327 genes) of the set of genes that where targeted at least once by an RNAi experiment. Among singleton genes (*i.e.*, genes with no paralogs) for which we have a phenotypic class, the fraction of PH genes is 19% (1,846 out of 9,943).

As previously described [11,31,32], the rate of false negatives in a single RNAi screen is around 50%, based on comparison of the RNAi results to the loss-of-function phenotypes caused by genetic mutations. Matching our RNAi data to the phenotypes described for 372 genetic mutants, we observed that the overall false negative rate falls to 37% when the union of all screens is considered. Using similar comparison analysis with genetic data, very low rates of false positives (less than 4%) were previously described [11,31,32] and were considered negligible in our study. Because the rate of false negatives in an RNAi assay is considerably higher than the rate of false positives, if a gene was described as PH in at least one RNAi assay, it will be considered PH for our purpose. In our analysis, both viable and lethal phenotypes were scored in an identical manner (PH genes).

### Local identity between the gene sequences and the RNAi clone sequences

After analyzing the overlapping positions of the genes and the RNAi clones on the genomic sequence, we identified 540 pairs of strict paralogs (gene families with only two members) for which both copies were targeted by one or more RNAi clones (see Additional file 1). For the genes that were targeted by more than one clone, we took the longest RNAi clone. For each pair of paralogs, we can determine "gene a" and its corresponding "RNAI clone a" as well as "gene b" and its corresponding "RNAi clone b". We calculated percentage identities between the sequences of 1) "gene a" and "RNAi clone b" and 2) "gene b" and "RNAi clone a" over lengths of 25 nt, 30 nt, 40 nt, 50 nt, 60 nt, 100 nt, 200 nt and 300 nt. For each pair and for each length, we determined the maximum percentage identity that it is possible to score between the two sequences (being "gene a" sequence versus "RNAi clone b" sequence or "gene b" sequence versus "RNAi clone a" sequence) and keep the lowest (this being more conservative). To determine the maximum percentage identity, we constructed all subsequences of the selected length from one gene and align each of these subsequences with the RNAi clone sequence of the other paralog. We used global alignment with no penalty for end gaps. An identity score matrix (+1 for matches and -1 for mismatches) was used with gap opening -4 and gap extension -1. In this scoring system, gaps are relatively rare. For each alignment, identity was calculated as the number of matches over the selected length (gaps do not count). To handle memory consumption, we excluded 16 pairs where at least one gene was larger than 5 kb from the analysis, leaving 540 - 16 = 524 pairs for the analysis of identity percent. The program was developed in C and sources are available upon request.

### A model of off-target cross-reaction

We elaborated a model in which a pair of duplicates systematically exhibits cross-reaction. The idea is to test whether this model fits well for pairs of duplicates where both copies have sequences very similar to the sequences of each other's RNAi clones. In this model, we make three important assumptions:

a) The probability that an ancestral gene that experienced a duplication event (and gave rise to a pair) was WT or PH is given by the frequency of WT and PH in singleton genes. We assume that at least one copy of the pair inherits the phenotypic class.

b) In the RNAi dataset, the rate of false negatives (genes scored as WT but which are PH) is 0.37. False positives are considered negligible (see above).

c) If one member of a pair has an RNAi phenotype (a PH gene), the other one systematically has a phenotype as well (due to off-target cross-reaction). Thus if there were no false negatives, we shall only see PH/PH or WT/WT pairs.

Based on this model, we can compute the expected frequencies of WT/WT, WT/PH and PH/PH pairs. To do so, we first compute the adjusted fraction of PH genes, after correction for the rate of false negatives, as 0.19/(1 - 0.37) = 0.30. Then, if we assume complete off-target cross-reaction, the fraction of real PH/PH pairs is also 0.30. It is to be noted that results were also obtained by considering fraction of real PH/PH pairs equal to 0.15 or 0.60 and that the conclusions of the analysis remain the same. Finally, since each PH gene of the pair has 0.37 chance to be considered negative in an RNAi assay, only (1 - 0.37) × (1 - 0.37) of the real PH/PH pairs will be scored so. This leads to an expected fraction of observed PH/PH pairs of (1 - 0.37) × (1 - 0.37) × 0.30 = 0.12. Similarly, we expect to observe 2 × (1 - 0.37) × 0.37 × 0.30 = 0.14 of WT/PH pairs and 1 - 0.12 - 0.14 = 0.74 of WT/WT pairs.

### Likelihood probability

Using the expected frequencies of PH/PH, WT/PH and WT/WT pairs, we computed the multinomial probability of having the exact observed number of pairs in each phenotypic category.

Mult(Nphph,Nphwt|freq)=(N!Nphph!Nphwt!(N−Nphph−Nphwt)!)×0.12Nphph×0.14Nphwt×0.74(N−Nphph−Nphwt),
 MathType@MTEF@5@5@+=feaafiart1ev1aaatCvAUfKttLearuWrP9MDH5MBPbIqV92AaeXatLxBI9gBaebbnrfifHhDYfgasaacH8akY=wiFfYdH8Gipec8Eeeu0xXdbba9frFj0=OqFfea0dXdd9vqai=hGuQ8kuc9pgc9s8qqaq=dirpe0xb9q8qiLsFr0=vr0=vr0dc8meaabaqaciaacaGaaeqabaqabeGadaaakeaacqWGnbqtcqWG1bqDcqWGSbaBcqWG0baDcqGGOaakcqWGobGtdaWgaaWcbaGaemiCaaNaemiAaGMaemiCaaNaemiAaGgabeaakiabcYcaSiabd6eaonaaBaaaleaacqWGWbaCcqWGObaAcqWG3bWDcqWG0baDaeqaaOGaeiiFaWNaemOzayMaemOCaiNaemyzauMaemyCaeNaeiykaKIaeyypa0ZaaeWaaeaadaWcaaqaaiabd6eaojabcgcaHaqaaiabd6eaonaaBaaaleaacqWGWbaCcqWGObaAcqWGWbaCcqWGObaAaeqaaOGaeiyiaeIaemOta40aaSbaaSqaaiabdchaWjabdIgaOjabdEha3jabdsha0bqabaGccqGGHaqicqGGOaakcqWGobGtcqGHsislcqWGobGtdaWgaaWcbaGaemiCaaNaemiAaGMaemiCaaNaemiAaGgabeaakiabgkHiTiabd6eaonaaBaaaleaacqWGWbaCcqWGObaAcqWG3bWDcqWG0baDaeqaaOGaeiykaKIaeiyiaecaaaGaayjkaiaawMcaaiabgEna0kabicdaWiabc6caUiabigdaXiabikdaYmaaCaaaleqabaGaemOta40aaSbaaWqaaiabdchaWjabdIgaOjabdchaWjabdIgaObqabaaaaOGaey41aqRaeGimaaJaeiOla4IaeGymaeJaeGinaqZaaWbaaSqabeaacqWGobGtdaWgaaadbaGaemiCaaNaemiAaGMaem4DaCNaemiDaqhabeaaaaGccqGHxdaTcqaIWaamcqGGUaGlcqaI3aWncqaI0aandaahaaWcbeqaaiabcIcaOiabd6eaojabgkHiTiabd6eaonaaBaaameaacqWGWbaCcqWGObaAcqWGWbaCcqWGObaAaeqaaSGaeyOeI0IaemOta40aaSbaaWqaaiabdchaWjabdIgaOjabdEha3jabdsha0bqabaWccqGGPaqkaaGccqGGSaalaaa@A454@

where *N *is the total number of observed pairs, *N*_*phph *_is the number PH/PH pairs and *N*_*wtph *_is the number WT/PH pairs. Expected frequencies were estimated using the above model of off-target cross-reaction. Because the total number of observation is not the same in all bins, we had to use cumulative probabilities. If off-target effect does not occur, we intuitively expect to observe more WT/PH pairs and less PH/PH pairs. Therefore, we chose to cumulate the probabilities in the following way:

P=FLikelihood(Data|freq)=∑i=0Nphph∑j=NwtphN−iMult(i,j|freq)
 MathType@MTEF@5@5@+=feaafiart1ev1aaatCvAUfKttLearuWrP9MDH5MBPbIqV92AaeXatLxBI9gBaebbnrfifHhDYfgasaacH8akY=wiFfYdH8Gipec8Eeeu0xXdbba9frFj0=OqFfea0dXdd9vqai=hGuQ8kuc9pgc9s8qqaq=dirpe0xb9q8qiLsFr0=vr0=vr0dc8meaabaqaciaacaGaaeqabaqabeGadaaakeaacqWGqbaucqGH9aqpcqWGgbGrdaWgaaWcbaGaemitaWKaemyAaKMaem4AaSMaemyzauMaemiBaWMaemyAaKMaemiAaGMaem4Ba8Maem4Ba8MaemizaqgabeaakiabcIcaOiabdseaejabdggaHjabdsha0jabdggaHjabcYha8jabdAgaMjabdkhaYjabdwgaLjabdghaXjabcMcaPiabg2da9maaqadabaWaaabmaeaacqWGnbqtcqWG1bqDcqWGSbaBcqWG0baDcqGGOaakcqWGPbqAcqGGSaalcqWGQbGAcqGG8baFcqWGMbGzcqWGYbGCcqWGLbqzcqWGXbqCcqGGPaqkaSqaaiabdQgaQjabg2da9iabd6eaonaaBaaameaacqWG3bWDcqWG0baDcqWGWbaCcqWGObaAaeqaaaWcbaGaemOta4KaeyOeI0IaemyAaKganiabggHiLdaaleaacqWGPbqAcqGH9aqpcqaIWaamaeaacqWGobGtdaWgaaadbaGaemiCaaNaemiAaGMaemiCaaNaemiAaGgabeaaa0GaeyyeIuoaaaa@790A@

One should mention that this cumulative likelihood probability relates to a one-tail statistical test. In that respect, the cumulative probability *P *will be higher than 0.5 if the observed counts exceed the expectations. In our particular case, if one observes too many PH/PH pairs or too few WT/PH pairs, it will give rise to a high probability. This is illustrated in Figure 1, when pairs having a local identity score lower than 100% show a better likelihood probability (for 50 nt, 100 nt, 200 nt and 300 nt). Nevertheless, this does not affect the loss of fit for data with medium identities.

## Authors' contributions

JFR and GA contributed equally to this work. The project was conceived and designed by JFR and GA. Computational analyses were performed by GA. RNAi data were collected from WormBase by NK. Analysis and overall interpretation of data were performed by JFR and GA. The manuscript was written by JFR and GA. All authors read and approved the final manuscript.

## Supplementary Material

Additional file 1 List of the 540 pairs of gene duplicates, their largest clones and their corresponding phenotypic classification Click here for file

## Figures and Tables

**Table 1:** Estimation of the minimum length of sequence similarity required in order to observe off-target cross-reaction

	**100% over 25 nt**	**100% over 30 nt**	**≥95% over 40 nt**	**≥94% over 50 nt**	**≥88% over 60 nt**
**<100% over 25 nt**	-	*N *= 0 *P *= n/a			
**<100% over 30 nt**	*N *= 21 *P *= 0.06	-	*N *= 25 *P *= 0.37		
**<95% over 40 nt**		*N *= 1 *P *= n/a	-	*N *= 1 *P *= n/a	
**<94% over 50 nt**			*N *= 13 *P *= 0.32	-	*N *= 42 *P *= 0.01
**<88% over 60 nt**				*N *= 0 *P *= n/a	

*N *is the number of pairs in a given cell and *P *the likelihood probability of being subject to off-target cross-reaction. We chose not to compute the likelihood probability when *N *was below 10 pairs. If the likelihood probability is high (*i.e. *in the vicinity of 0.3), the fractions of WT/WT, WT/PH and PH/PH for the selected pairs do fit our model (see text) and are therefore likely to be subject to off-target cross-reaction. From this table, we predict that efficient RNAi is observed for pairs sharing high percentage identity over more than 25 nt and less than 60 nt. High identity on 40 nt (without high identity on 30 nt or 50 nt) fits well with efficient RNAi, however since the data used here are limited, we think it is reasonable to propose that RNAi is maximized when sequence similarity is very high in the range 30–50 nt. We are aware that the counts we used here can be sometimes low; in the future, the use of a larger RNAi data set should allow to gain in statistical power.
