# Supplementary material for: Novel insights into RNAi off-target effects using C. elegans paralogs
Source: BMC Genomics. 2007 Apr 19;8:106. doi: 10.1186/1471-2164-8-106 (PMC1868761; doi:10.1186/1471-2164-8-106)
Supplement: Additional file 1 — List of the 540 pairs of gene duplicates, their largest clones and their corresponding phenotypic classification [file 1471-2164-8-106-S1.pdf]

SUPPLEMENTARY TABLE

Table S1: List of the 540 pairs of gene duplicates, their largest clone and their corresponding phenotypic classification.

Paralog1	Paralog2	Largest Clone of P. 1	Largest Clone of P. 2	Phenotype of P.1	Phenotype of P.2
AC8.6	Y110A7A.19	mv_AC8.6	mv_Y110A7A.19	PH	PH
B0205.6	F13H8.9	mv_B0205.6	mv_F13H8.9	PH	PH
B0207.4	K07C11.2	cgc4544_air_2	mv_K07C11.2	PH	PH
B0207.6	Y75B8A.14	mv_B0207.6	mv_Y75B8A.14	PH	PH
C04C3.3	F27D4.5	mv_C04C3.3	mv_F27D4.5	PH	PH
C05C12.3	T01H8.5	mv_C05C12.3	mv_T01H8.5	PH	PH
C25H3.8	R12C12.8	mv_C25H3.8	mv_R12C12.8	PH	PH
C26E6.4	F09F7.3	mv_C26E6.4	mv_F09F7.3	PH	PH
C27H6.2	T22D1.10	mv_C27H6.2	mv_T22D1.10	PH	PH
C32F10.5	T20B12.8	mv_C32F10.5	mv_T20B12.8	PH	PH
C37C3.3	C56C10.3	mv_C37C3.3	mv_C56C10.3	PH	PH
C38C10.4	F22B7.13	mv_C38C10.4	mv_F22B7.13	PH	PH
C43E11.4	Y71H2AM.23	JA_C43E11.4	JA_Y71H2_378.a	PH	PH
C54C6.1	W01D2.1	mv_C54C6.1	Simmer_W01D2.1	PH	PH
F01G12.5	K04H4.1	JA_F01G12.5a	JA_K04H4.1	PH	PH
F07A11.2	F22B3.4	mv_F07A11.2	mv_F22B3.4	PH	PH
F09E5.2	T23F2.1	mv_F09E5.2	mv_T23F2.1	PH	PH
F20A1.9	Y55F3BR.1	mv_F20A1.9	JA_C44C8.5	PH	PH
F25B5.4	ZK1010.1	mv_F25B5.4	JA_ZK1010.1	PH	PH
F25H5.4	ZK328.2	JA_F25H5.4	mv_ZK328.2	PH	PH
F26B1.3	F32E10.4	mv_F26B1.3	JA_F32E10.4	PH	PH
F32E10.6	Y55B1BR.3	mv_F32E10.6	mv_Y55B1BR.3	PH	PH
F32H2.5	F32H2.6	mv_F32H2.5	mv_F32H2.6	PH	PH
F52B5.6	F55D10.2	mv_F52B5.6	mv_F55D10.2	PH	PH
F53B2.1	R11A8.2	mv_F53B2.1	mv_R11A8.2	PH	PH
F53G12.3	F56C11.1	mv_F53G12.3	mv_F56C11.1	PH	PH
F54C8.2	F58A4.3	mv_F54C8.2	JA_F58A4.3	PH	PH
F54C9.1	T05G5.10	mv_F54C9.1	mv_T05G5.10	PH	PH
F56B3.2	M03F4.6	mv_F56B3.2	mv_M03F4.6	PH	PH
F59E12.4	F59E12.5	mv_F59E12.4	mv_F59E12.5	PH	PH
K07C5.4	W01B11.3	mv_K07C5.4	mv_W01B11.3	PH	PH
K07D8.1	K08E5.3	mv_K07D8.1	mv_K08E5.3	PH	PH
K09A9.5	T26A5.3	mv_K09A9.5	mv_T26A5.3	PH	PH
K09H11.3	Y75B7AL.4	mv_K09H11.3	SA_yk146f7	PH	PH
R12E2.10	T21E3.1	mv_R12E2.10	mv_T21E3.1	PH	PH
T16H12.5	ZK858.4	mv_T16H12.5	JA_ZK858.4	PH	PH

Y23H5A.1	Y23H5A.7	mv_Y23H5A.1	mv_Y23H5A.7	PH	PH
Y87G2A.5	ZC513.4	mv_Y87G2A.5	mv_ZC513.4	PH	PH
B0244.8	R01H2.3	mv_B0244.8	mv_R01H2.3	PH	WT
B0250.7	T10G3.5	JA_B0250.7	mv_T10G3.5	PH	WT
B0280.1	Y48E1B.3	mv_B0280.1.v5	mv_Y48E1B.3	PH	WT
C01F1.3	F53B1.4	mv_C01F1.3	mv_F53B1.4	PH	WT
C01G5.2	D2030.6	mv_C01G5.2	mv_D2030.6	PH	WT
C01G8.5	F42A10.2	mv_C01G8.5a	mv_F42A10.2	PH	WT
C04F6.4	K08F9.2	mv_C04F6.4	mv_K08F9.2	PH	WT
C05C8.7	ZK632.4	mv_C05C8.7	mv_ZK632.4	PH	WT
C27A2.6	C34F11.9	mv_C27A2.6	mv_C34F11.9a	PH	WT
C27H5.4	F10E7.3	mv_C27H5.4	mv_F10E7.3	PH	WT
C29F9.8	C29F9.9	mv_C29F9.8	mv_C29F9.9	PH	WT
C31C9.2	F49E10.5	mv_C31C9.2	mv_F49E10.5	PH	WT
C33C12.4	Y54G2A.15	mv_C33C12.4	mv_Y54G2A.15	PH	WT
C40H5.6	K03B8.4	mv_C40H5.6	mv_K03B8.4	PH	WT
C42C1.5	Y47D9A.1	mv_C42C1.5	mv_Y47D9A.1a	PH	WT
C48A7.1	T02C5.5	JA_C48A7.1	mv_T02C5.5	PH	WT
C50F7.4	F47B10.1	mv_C50F7.4	JA_F47B10.1	PH	WT
C54D1.5	F41C6.1	mv_C54D1.5	JA_F41C6.1	PH	WT
C54F6.14	D1037.3	mv_C54F6.14	JA_D1037.3	PH	WT
C54H2.5	T02E1.7	JA_C54H2.5	mv_T02E1.7	PH	WT
D2062.4	D2062.5	mv_D2062.4	mv_D2062.5	PH	WT
E04A4.7	ZC116.2	mv_E04A4.7	mv_ZC116.2	PH	WT
F01F1.11	Y47H9C.8	mv_F01F1.11	mv_Y47H9C.8	PH	WT
F02A9.4	F52E4.1	mv_F02A9.4b	mv_F52E4.1	PH	WT
F08B6.4	T25F10.6	JA_F08B6.4	mv_T25F10.6	PH	WT
F09C3.4	T06E6.13	mv_F09C3.4	mv_T06E6.13	PH	WT
F10B5.3	F40F8.7	mv_F10B5.3	JA_F40F8.7	PH	WT
F20B6.2	Y110A7A.12	mv_F20B6.2	mv_Y110A7A.12	PH	WT
F25H5.3	ZK593.1	mv_F25H5.3a	mv_ZK593.1	PH	WT
F26H9.4	W06B3.1	mv_F26H9.4	mv_W06B3.1	PH	WT
F28H1.2	F49D11.8	mv_F28H1.2	mv_F49D11.8	PH	WT
F32B5.8	M04G12.2	mv_F32B5.8	mv_M04G12.2	PH	WT
F39B2.10	T05C3.5	mv_F39B2.10	mv_T05C3.5	PH	WT
F40F9.6	F52D1.1	mv_F40F9.6a	mv_F52D1.1	PH	WT
F45H10.2	R07E4.3	mv_F45H10.2	mv_R07E4.3	PH	WT
F49H12.1	F52F12.4	mv_F49H12.1	JA_F52F12.4	PH	WT
F54D1.6	R09E10.5	mv_F54D1.6	mv_R09E10.5	PH	WT
F55A12.3	Y48G9A.8	mv_F55A12.3	mv_Y48G9A.8	PH	WT
F56D2.1	ZC410.2	mv_F56D2.1	mv_ZC410.2	PH	WT
F57B9.10	F59B2.5	mv_F57B9.10	mv_F59B2.5	PH	WT
H06I04.3	R74.7	mv_H06I04.3a	mv_R74.7.v5	PH	WT
K02A4.1	Y44A6D.5	JA_K02A4.1	mv_Y44A6D.5	PH	WT
K08D10.3	K08D10.4	mv_K08D10.3	mv_K08D10.4	PH	WT
K09E4.1	Y111B2A.27	mv_K09E4.1	JA_Y111B2C.f	PH	WT
K12D12.3	W08D2.6	mv_K12D12.3	mv_W08D2.6	PH	WT

R12E2.11	T07C4.1	mv_R12E2.11	mv_T07C4.1	PH	WT
R151.2	W04G3.5	mv_R151.2	mv_W04G3.5	PH	WT
R186.1	Y80D3A.9	mv_R186.1	mv_Y80D3A.9	PH	WT
T04G9.4	T28H10.1	mv_T04G9.4	mv_T28H10.1	PH	WT
T06E4.1	ZK1055.1	mv_T06E4.1	mv_ZK1055.1	PH	WT
T09B4.2	Y37A1B.14	mv_T09B4.2	mv_Y37A1B.14	PH	WT
T23B3.4	Y39A3B.5	mv_T23B3.4	mv_Y39A3B.5	PH	WT
W09C5.2	Y50E8A.4	mv_W09C5.2	mv_Y50E8A.4	PH	WT
Y47G6A.12	ZK430.5	mv_Y47G6A.12	mv_ZK430.5	PH	WT
Y48B6A.3	Y53F4B.6	mv_Y48B6A.3	mv_Y53F4B.6	PH	WT
Y49E10.20	Y76A2B.6	mv_Y49E10.20	mv_Y76A2B.6	PH	WT
Y57A10A.10	Y57A10A.28	mv_Y57A10A.10	mv_Y57A10A.28	PH	WT
Y65B4A.9	ZK354.2	JA_Y65B4A_185.a	mv_ZK354.2	PH	WT
Y65B4BR.4	Y92H12A.2	mv_Y65B4BR.4	SA_yk208a4	PH	WT
Y73F4A.2	Y73F4A.3	mv_Y73F4A.2	mv_Y73F4A.3	PH	WT
ZK507.1	ZK507.3	mv_ZK507.1	mv_ZK507.3	PH	WT
B0001.4	F19B6.1	mv_B0001.4	mv_F19B6.1b	WT	PH
B0024.12	T23G11.2	JA_B0024.12	mv_T23G11.2	WT	PH
B0198.3	F54F7.5	mv_B0198.3	mv_F54F7.5	WT	PH
C09H10.6	C50B6.2	mv_C09H10.6	mv_C50B6.2	WT	PH
C10C6.3	C15H7.4	mv_C10C6.3	mv_C15H7.4	WT	PH
C17D12.2	T01D1.2	mv_C17D12.2	mv_T01D1.2a	WT	PH
C18B2.4	D2013.6	JA_C18B2.4	mv_D2013.6	WT	PH
C23H4.6	F54D5.14	mv_C23H4.6	mv_F54D5.14	WT	PH
C24H12.2	C24H12.5	mv_C24H12.2	mv_C24H12.5	WT	PH
C26D10.4	C26D10.5	mv_C26D10.4	mv_C26D10.5	WT	PH
C30G12.7	W06B11.2	mv_C30G12.7	mv_W06B11.2	WT	PH
C44C8.6	K08F8.1	mv_C44C8.6	mv_K08F8.1	WT	PH
C44H4.6	Y18D10A.5	mv_C44H4.6	mv_Y18D10A.5	WT	PH
C46H11.3	M01B12.3	mv_C46H11.3	mv_M01B12.3	WT	PH
C49F5.3	F02D10.6	mv_C49F5.3	mv_F02D10.6	WT	PH
C53A5.13	H19M22.2	mv_C53A5.13	JA_H19M22.2	WT	PH
C53B4.7	F56H6.5	mv_C53B4.7	mv_F56H6.5	WT	PH
C53B7.4	K07A12.3	mv_C53B7.4	mv_K07A12.3	WT	PH
D1005.2	K08E3.5	mv_D1005.2	mv_K08E3.5b	WT	PH
D1025.2	F52A8.5	mv_D1025.2	mv_F52A8.5	WT	PH
E02H4.6	K04C1.5	mv_E02H4.6	mv_K04C1.5	WT	PH
E02H9.8	H10E21.3	mv_E02H9.8	mv_H10E21.3	WT	PH
F02C9.3	F36H2.1	mv_F02C9.3	mv_F36H2.1	WT	PH
F02E8.3	F29G9.3	mv_F02E8.3	mv_F29G9.3	WT	PH
F09D12.1	F41E6.2	mv_F09D12.1	mv_F41E6.2	WT	PH
F17E5.2	F55A11.4	mv_F17E5.2	mv_F55A11.4	WT	PH
F20D6.8	W04C9.5	mv_F20D6.8	mv_W04C9.5	WT	PH
F21A10.2	F59B10.1	mv_F21A10.2	mv_F59B10.1	WT	PH
F22B5.4	F36A2.7	mv_F22B5.4	mv_F36A2.7	WT	PH
F28F8.2	F46E10.1	mv_F28F8.2	JA_F46E10.1	WT	PH
F33H2.3	T19H12.2	mv_F33H2.3	mv_T19H12.2	WT	PH

F35C8.6	Y18D10A.20	mv_F35C8.6	mv_Y18D10A.20	WT	PH
F37A4.5	K07D4.3	mv_F37A4.5	mv_K07D4.3	WT	PH
F37B4.2	M6.1	JA_F37B4.2	JA_M6.1	WT	PH
F40E10.2	K08A8.2	mv_F40E10.2	mv_K08A8.2	WT	PH
F40F4.1	H24O09.2	mv_F40F4.1	mv_H24O09.2	WT	PH
F42G9.8	Y111B2A.15	mv_F42G9.8	SA_yk363g6	WT	PH
F52C12.5	F55A8.1	mv_F52C12.5	mv_F55A8.1	WT	PH
F52C6.2	F52C6.3	mv_F52C6.2	mv_F52C6.3	WT	PH
F53F8.1	F54H5.4	mv_F53F8.1	mv_F54H5.4	WT	PH
K08C9.1	K08C9.2	mv_K08C9.1	mv_K08C9.2	WT	PH
K12H6.6	Y63D3A.8	mv_K12H6.6	mv_Y63D3A.8	WT	PH
T08D2.7	Y60A3A.12	mv_T08D2.7	mv_Y60A3A.12	WT	PH
T28B8.3	T28B8.4	mv_T28B8.3	mv_T28B8.4	WT	PH
T28F3.5	W09B6.1	mv_T28F3.5	mv_W09B6.1	WT	PH
W04G3.3	W04G3.8	mv_W04G3.3	mv_W04G3.8	WT	PH
Y54G11A.4	Y54G11A.7	mv_Y54G11A.4	mv_Y54G11A.7	WT	PH
ZK112.1	ZK688.6	mv_ZK112.1	mv_ZK688.6	WT	PH
3R5.2	K08E3.8	JA_3R5.2	mv_K08E3.8	WT	WT
AC8.1	Y71F9AL.18	mv_AC8.1	JA_Y71F9A_270.b	WT	WT
B0019.2	F28H6.4	mv_B0019.2	mv_F28H6.4	WT	WT
B0035.2	C47A4.1	JA_B0035.2	JA_C47A4.1	WT	WT
B0035.6	Y111B2A.3	mv_B0035.6	JA_Y111B2A.b	WT	WT
B0212.2	T13F2.5	mv_B0212.2	mv_T13F2.5	WT	WT
B0272.4	R06F6.9	mv_B0272.4	mv_R06F6.9	WT	WT
B0273.1	B0334.10	mv_B0273.1	mv_B0334.10	WT	WT
B0280.4	C34H3.2	mv_B0280.4	mv_C34H3.2	WT	WT
B0303.2	T07C12.9	mv_B0303.2	mv_T07C12.9	WT	WT
B0304.5	B0304.6	mv_B0304.5	mv_B0304.6	WT	WT
B0361.11	C06H5.6	TH_302C6	mv_C06H5.6	WT	WT
B0365.1	D1005.1	mv_B0365.1	mv_D1005.1	WT	WT
B0403.4	Y49E10.4	mv_B0403.4	mv_Y49E10.4	WT	WT
B0416.2	ZK856.6	mv_B0416.2	mv_ZK856.6	WT	WT
B0432.2	C49G7.11	mv_B0432.2	mv_C49G7.11	WT	WT
B0454.6	F23F1.6	mv_B0454.6	mv_F23F1.6	WT	WT
B0454.8	B0554.4	mv_B0454.8	mv_B0554.4	WT	WT
B0496.6	C36H8.1	mv_B0496.6	mv_C36H8.1	WT	WT
B0496.7	F20D12.5	mv_B0496.7	JA_F20D12.5	WT	WT
B0511.4	Y38H8A.4	mv_B0511.4	mv_Y38H8A.4	WT	WT
B0513.1	C04B4.1	mv_B0513.1	mv_C04B4.1	WT	WT
B0524.2	D1054.1	mv_B0524.2	mv_D1054.1	WT	WT
B0564.7	Y75B8A.10	mv_B0564.7	mv_Y75B8A.10	WT	WT
C01B10.1	F15B10.2	mv_C01B10.1	mv_F15B10.2	WT	WT
C01B12.4	W01D2.5	mv_C01B12.4	mv_W01D2.5	WT	WT
C01B7.6	F07B7.12	mv_C01B7.6	SA_yk238a8	WT	WT
C01G12.3	F44G4.5	mv_C01G12.3	mv_F44G4.5	WT	WT
C01G5.4	C09B9.7	mv_C01G5.4	mv_C09B9.7	WT	WT
C02B8.2	F31A9.4	mv_C02B8.2	mv_F31A9.4	WT	WT

C02E7.13	C31A11.9	mv_C02E7.13	mv_C31A11.9	WT	WT
C03D6.5	F10G7.3	mv_C03D6.5	mv_F10G7.3	WT	WT
C03G6.7	C32B5.2	mv_C03G6.7	mv_C32B5.2	WT	WT
C04B4.2	C04B4.4	mv_C04B4.2	mv_C04B4.4	WT	WT
C04F5.7	C07G3.9	mv_C04F5.7	JA_C07G3.9	WT	WT
C04H5.2	EEED8.11	mv_C04H5.2	mv_EEED8.11	WT	WT
C05B5.4	R10E12.2	mv_C05B5.4	mv_R10E12.2	WT	WT
C05B5.7	F16H9.1	mv_C05B5.7	mv_F16H9.1a	WT	WT
C05D12.2	Y19D10B.4	mv_C05D12.2	mv_Y19D10B.4	WT	WT
C05E11.4	C05E11.5	mv_C05E11.4	mv_C05E11.5	WT	WT
C05E4.9	C08F11.14	JA_C05E4.9	mv_C08F11.14	WT	WT
C05E7.1	C05E7.2	mv_C05E7.1a	mv_C05E7.2	WT	WT
C06A12.3	Y43F8B.2	mv_C06A12.3	mv_Y43F8B.2a	WT	WT
C06A5.6	ZK856.12	mv_C06A5.6	mv_ZK856.12	WT	WT
C06A5.8	C06A5.9	mv_C06A5.8	mv_C06A5.9	WT	WT
C06A6.5	C30H7.2	mv_C06A6.5	mv_C30H7.2	WT	WT
C06A8.3	ZK970.7	mv_C06A8.3	mv_ZK970.7	WT	WT
C06B3.11	T10C6.3	mv_C06B3.11	mv_T10C6.3	WT	WT
C06B3.6	F13A7.11	mv_C06B3.6	mv_F13A7.11	WT	WT
C06E8.3	F45H7.4	mv_C06E8.3	mv_F45H7.4	WT	WT
C07G3.2	T24A6.7	mv_C07G3.2	mv_T24A6.7	WT	WT
C08A9.9	K09E3.5	mv_C08A9.9	mv_K09E3.5	WT	WT
C08F11.12	Y45F10C.2	mv_C08F11.12	JA_Y45F10C.2	WT	WT
C09B7.2	C09H10.5	mv_C09B7.2	mv_C09H10.5	WT	WT
C09B8.4	T10B11.6	mv_C09B8.4	mv_T10B11.6	WT	WT
C09F9.1	F38A5.8	mv_C09F9.1	mv_F38A5.8	WT	WT
C09G12.5	K03D3.5	mv_C09G12.5	mv_K03D3.5	WT	WT
C09G4.1	K02G10.6	mv_C09G4.1	mv_K02G10.6	WT	WT
C09G5.7	C54G4.2	mv_C09G5.7	mv_C54G4.2	WT	WT
C09H5.9	F58G4.5	mv_C09H5.9	mv_F58G4.5	WT	WT
C10G8.5	ZC168.1	JA_C10G8.5	mv_ZC168.1	WT	WT
C11D2.6	C27F2.2	mv_C11D2.6	mv_C27F2.2	WT	WT
C11E4.3	C46A5.2	mv_C11E4.3	mv_C46A5.2	WT	WT
C11G6.2	Y116F11A.1	mv_C11G6.2	mv_Y116F11A.1	WT	WT
C13B7.4	Y45G12C.9	mv_C13B7.4	mv_Y45G12C.9	WT	WT
C13C4.4	K08B12.3	mv_C13C4.4	mv_K08B12.3	WT	WT
C13C4.6	C27A7.6	mv_C13C4.6	mv_C27A7.6	WT	WT
C14B9.3	F36H9.5	mv_C14B9.3	mv_F36H9.5	WT	WT
C14C11.2	ZC317.7	JA_C14C11.2	mv_ZC317.7	WT	WT
C14C11.6	Y38A10A.6	mv_C14C11.6	mv_Y38A10A.6	WT	WT
C14E2.6	H41C03.2	mv_C14E2.6	mv_H41C03.2	WT	WT
C14F11.3	ZC504.5	mv_C14F11.3	mv_ZC504.5	WT	WT
C15B12.5	F47D12.1	JA_C15B12.5	mv_F47D12.1	WT	WT
C15C6.2	R09E10.6	mv_C15C6.2b	mv_R09E10.6	WT	WT
C16A11.4	Y14H12B.2	mv_C16A11.4	JA_Y14H12B.2	WT	WT
C16A11.7	F32B6.4	mv_C16A11.7	mv_F32B6.4	WT	WT
C17E7.8	C49D10.2	mv_C17E7.8	mv_C49D10.2	WT	WT

C17H11.6	Y49F6B.9	mv_C17H11.6	JA_Y49F6B.e	WT	WT
C18B12.5	H03G16.4	mv_C18B12.5	mv_H03G16.4	WT	WT
C18E3.8	F35H12.3	mv_C18E3.8	JA_F35H12.3	WT	WT
C24D10.2	F11G11.4	mv_C24D10.2	mv_F11G11.4	WT	WT
C25A8.2	R02D5.7	mv_C25A8.2	mv_R02D5.7	WT	WT
C25F6.4	F11D5.3	mv_C25F6.4	mv_F11D5.3	WT	WT
C25F9.1	C25F9.7	mv_C25F9.1	mv_C25F9.7	WT	WT
C25G4.4	C44F1.2	mv_C25G4.4	mv_C44F1.2	WT	WT
C25G4.7	ZK973.8	mv_C25G4.7	mv_ZK973.8	WT	WT
C25H3.14	C25H3.3	JA_C25H3.3	mv_C25H3.3	WT	WT
C26B2.2	ZK930.7	mv_C26B2.2	mv_ZK930.7	WT	WT
C27A7.7	VC27A7L.1	mv_C27A7.7	mv_VC27A7L.1	WT	WT
C27C12.2	Y55F3AM.7	mv_C27C12.2	mv_Y55F3AM.7	WT	WT
C27C12.7	T23F1.7	mv_C27C12.7	mv_T23F1.7a	WT	WT
C27F2.6	F17C8.5	mv_C27F2.6	mv_F17C8.5	WT	WT
C28A5.1	C28A5.2	mv_C28A5.1	mv_C28A5.2	WT	WT
C28A5.6	K09C6.1	mv_C28A5.6	mv_K09C6.1	WT	WT
C28H8.4	F09B9.3	mv_C28H8.4	JA_F09B9.3	WT	WT
C29F7.3	F40F8.1	mv_C29F7.3	mv_F40F8.1	WT	WT
C29F7.6	F23D12.5	mv_C29F7.6	mv_F23D12.5	WT	WT
C31G12.1	W04E12.2	mv_C31G12.1	mv_W04E12.2	WT	WT
C31H2.4	T21C12.2	mv_C31H2.4	mv_T21C12.2	WT	WT
C32E12.2	R08D7.6	mv_C32E12.2	mv_R08D7.6	WT	WT
C32H11.7	Y38F2AL.6	mv_C32H11.7	JA_Y38F2A_5743.c	WT	WT
C33D12.2	R04E5.2	mv_C33D12.2	mv_R04E5.2	WT	WT
C33E10.10	T25G12.2	mv_C33E10.10	mv_T25G12.2	WT	WT
C33F10.12	T05F1.8	mv_C33F10.12	mv_T05F1.8	WT	WT
C33H5.6	C33H5.7	mv_C33H5.6	mv_C33H5.7	WT	WT
C34B2.4	F33D11.1	mv_C34B2.4	mv_F33D11.1	WT	WT
C35A11.3	F49C5.7	mv_C35A11.3	mv_F49C5.7	WT	WT
C35C5.3	F28H7.4	mv_C35C5.3a	mv_F28H7.4	WT	WT
C35E7.9	F36H12.3	mv_C35E7.9	mv_F36H12.3	WT	WT
C36A4.8	F42A6.5	mv_C36A4.8	mv_F42A6.5	WT	WT
C36B1.11	Y48G10A.2	mv_C36B1.11	mv_Y48G10A.2	WT	WT
C36C9.2	ZK380.1	mv_C36C9.2	JA_Y75D11B.a	WT	WT
C37H5.10	C37H5.11	mv_C37H5.10	mv_C37H5.11	WT	WT
C38C3.7	Y40B10A.4	mv_C38C3.7	JA_Y40B10A.f	WT	WT
C38C6.2	F26D10.9	mv_C38C6.2	mv_F26D10.9	WT	WT
C38D4.4	F58G11.3	mv_C38D4.4	mv_F58G11.3	WT	WT
C39D10.3	Y37E3.11	mv_C39D10.3	SA_yk299e12	WT	WT
C39E6.6	T05A1.1	mv_C39E6.6	mv_T05A1.1	WT	WT
C39E9.8	Y105C5A.8	mv_C39E9.8	mv_Y105C5A.8	WT	WT
C40A11.5	K09F6.3	mv_C40A11.5	mv_K09F6.3	WT	WT
C40C9.1	F34D6.3	mv_C40C9.1	JA_F34D6.3	WT	WT
C41G6.2	M01B2.11	mv_C41G6.2	JA_C41G6.2	WT	WT
C42D8.4	T08H4.3	mv_C42D8.4	mv_T08H4.3	WT	WT
C43D7.4	C43D7.5	mv_C43D7.4	JA_C43D7.5	WT	WT

C44B12.4	Y55F3AM.2	mv_C44B12.4	mv_Y55F3AM.2	WT	WT
C44C1.2	R09B5.12	mv_C44C1.2	mv_R09B5.12	WT	WT
C44C11.2	C50E10.4	mv_C44C11.2	mv_C50E10.4	WT	WT
C44H4.1	ZK682.5	mv_C44H4.1	mv_ZK682.5	WT	WT
C45B11.4	T04C12.2	mv_C45B11.4	mv_T04C12.2	WT	WT
C45G9.4	C45G9.9	mv_C45G9.4	mv_C45G9.9	WT	WT
C46E10.4	C46E10.5	mv_C46E10.4	mv_C46E10.5	WT	WT
C46E10.9	F12E12.5	mv_C46E10.9	mv_F12E12.5	WT	WT
C46H11.6	F28E10.4	mv_C46H11.6	mv_F28E10.4	WT	WT
C47B2.1	F08A8.7	mv_C47B2.1	mv_F08A8.7	WT	WT
C47C12.4	Y47G6A.27	JA_C47C12.4	JA_Y47G6A_245.j	WT	WT
C47D2.2	F49E8.4	mv_C47D2.2	mv_F49E8.4	WT	WT
C47E12.11	F02E9.3	mv_C47E12.11	mv_F02E9.3	WT	WT
C48E7.7	T08B2.12	mv_C48E7.7	mv_T08B2.12	WT	WT
C49C3.6	C49C3.7	mv_C49C3.6	mv_C49C3.7	WT	WT
C49G7.9	F52F10.1	mv_C49G7.9	mv_F52F10.1	WT	WT
C50E3.7	Y75B8A.34	mv_C50E3.7	mv_Y75B8A.34	WT	WT
C50F7.10	E02H9.5	mv_C50F7.10	mv_E02H9.5	WT	WT
C50H11.1	F41C3.3	mv_C50H11.1	mv_F41C3.3	WT	WT
C50H2.4	C50H2.5	mv_C50H2.4	mv_C50H2.5	WT	WT
C52A11.3	C52A11.4	mv_C52A11.3	mv_C52A11.4	WT	WT
C53B4.6	F15B10.1	mv_C53B4.6	JA_F15B10.1	WT	WT
C53B7.7	F40B5.1	mv_C53B7.7	mv_F40B5.1	WT	WT
C53C11.3	Y65B4BR.3	mv_C53C11.3	mv_Y65B4BR.3	WT	WT
C54D1.2	Y38H6C.8	mv_C54D1.2	mv_Y38H6C.8	WT	WT
C54F6.10	T27C4.3	mv_C54F6.10	mv_T27C4.3	WT	WT
C54F6.11	C54F6.6	mv_C54F6.11	mv_C54F6.6	WT	WT
C54G10.3	T10H9.5	mv_C54G10.3	mv_T10H9.5	WT	WT
C55A1.9	ZK380.4	mv_C55A1.9	mv_ZK380.4	WT	WT
C55A6.1	Y105E8A.14	mv_C55A6.1	JA_Y105E8C.f	WT	WT
D1005.3	Y44E3B.1	mv_D1005.3	mv_Y44E3B.1	WT	WT
D1014.8	Y74C9A.4	mv_D1014.8	mv_Y74C9A.4	WT	WT
D1022.8	F54D8.4	JA_D1022.8	mv_F54D8.4	WT	WT
D1037.5	H23L24.2	mv_D1037.5	mv_H23L24.2	WT	WT
D1044.1	H06H21.8	mv_D1044.1	mv_H06H21.8	WT	WT
D1065.4	D1065.5	mv_D1065.4	mv_D1065.5	WT	WT
D1081.5	F54H5.5	mv_D1081.5	mv_F54H5.5	WT	WT
D2024.2	Y45F10A.3	mv_D2024.2	mv_Y45F10A.3	WT	WT
D2030.2	K07A3.3	mv_D2030.2	mv_K07A3.3	WT	WT
D2045.2	H04D03.3	mv_D2045.2	mv_H04D03.3	WT	WT
D2096.6	T25E4.1	mv_D2096.6	mv_T25E4.1	WT	WT
E02H4.4	K06G5.1	mv_E02H4.4	mv_K06G5.1	WT	WT
E04F6.8	E04F6.9	mv_E04F6.8	mv_E04F6.9	WT	WT
F01D5.10	F25E5.2	mv_F01D5.10	mv_F25E5.2	WT	WT
F02H6.2	K01G12.3	mv_F02H6.2	mv_K01G12.3	WT	WT
F07C3.3	M03F8.5	mv_F07C3.3	mv_M03F8.5	WT	WT
F07E5.8	T16A1.2	mv_F07E5.8	mv_T16A1.2	WT	WT

F08D12.2	F08D12.3	mv_F08D12.2	mv_F08D12.3	WT	WT
F08F8.1	F08F8.5	mv_F08F8.1	mv_F08F8.5	WT	WT
F09E10.8	K08E3.3	mv_F09E10.8	mv_K08E3.3a	WT	WT
F10D11.5	T15H9.4	mv_F10D11.5	mv_T15H9.4	WT	WT
F10D2.3	T07H8.7	mv_F10D2.3	mv_T07H8.7	WT	WT
F10G8.4	Y44A6D.4	mv_F10G8.4	mv_Y44A6D.4	WT	WT
F11A5.1	F11A5.2	mv_F11A5.1	mv_F11A5.2	WT	WT
F11C1.1	F42H10.2	mv_F11C1.1	mv_F42H10.2	WT	WT
F11C1.4	F26A3.1	mv_F11C1.4	mv_F26A3.1	WT	WT
F11E6.7	F19H6.3	mv_F11E6.7	mv_F19H6.3	WT	WT
F12A10.2	ZK177.2	mv_F12A10.2	mv_ZK177.2	WT	WT
F12B6.2	ZK682.2	mv_F12B6.2	mv_ZK682.2	WT	WT
F13A7.3	F57G8.8	mv_F13A7.3	mv_F57G8.8	WT	WT
F13D12.5	T21B10.4	mv_F13D12.5	mv_T21B10.4	WT	WT
F13H8.3	Y43F8C.13	mv_F13H8.3	mv_Y43F8C.13	WT	WT
F14F4.1	T07D10.2	mv_F14F4.1	mv_T07D10.2	WT	WT
F14F8.2	F14F8.3	mv_F14F8.2	mv_F14F8.3	WT	WT
F14F9.7	Y47G7B.1	mv_F14F9.7	mv_Y47G7B.1	WT	WT
F14H8.4	F14H8.5	mv_F14H8.4	mv_F14H8.5	WT	WT
F15B10.3	F38A5.7	mv_F15B10.3	mv_F38A5.7	WT	WT
F15E6.1	Y51H4A.12	mv_F15E6.1	JA_Y51H4A.l	WT	WT
F16B4.5	F16B4.7	mv_F16B4.5	mv_F16B4.7	WT	WT
F19F10.1	F19F10.5	mv_F19F10.1	mv_F19F10.5	WT	WT
F19G12.1	T13A10.2	JA_F19G12.1	mv_T13A10.2	WT	WT
F19H8.1	ZK54.2	mv_F19H8.1	mv_ZK54.2.v5	WT	WT
F20C5.4	R08C7.2	mv_F20C5.4	mv_R08C7.2	WT	WT
F20D1.4	F45E4.2	mv_F20D1.4	mv_F45E4.2	WT	WT
F21F8.1	T21C9.7	mv_F21F8.1	mv_T21C9.7	WT	WT
F21G4.5	K04C1.3	mv_F21G4.5	JA_K04C1.3	WT	WT
F23A7.6	T06G6.5	mv_F23A7.6	mv_T06G6.5	WT	WT
F23H11.1	F54B11.6	mv_F23H11.1	mv_F54B11.6	WT	WT
F25D1.1	T23F11.1	mv_F25D1.1	mv_T23F11.1	WT	WT
F25E2.4	R04E5.10	mv_F25E2.4	mv_R04E5.10	WT	WT
F25E5.4	F25E5.7	mv_F25E5.4	mv_F25E5.7	WT	WT
F25G6.6	M02D8.4	mv_F25G6.6	mv_M02D8.4	WT	WT
F25H5.7	F55F8.7	mv_F25H5.7	mv_F55F8.7	WT	WT
F26A1.4	ZK354.6	mv_F26A1.4	mv_ZK354.6	WT	WT
F26F4.2	F37A8.1	mv_F26F4.2	mv_F37A8.1	WT	WT
F26H9.8	F48E3.3	mv_F26H9.8	mv_F48E3.3	WT	WT
F28A12.3	F35C5.11	mv_F28A12.3	mv_F35C5.11	WT	WT
F28B4.3	F40F4.6	mv_F28B4.3	mv_F40F4.6	WT	WT
F28F8.7	Y73B3A.13	mv_F28F8.7	TH_304F11_13	WT	WT
F29B9.2	F43G6.6	mv_F29B9.2	mv_F43G6.6	WT	WT
F31C3.2	K05C4.4	mv_F31C3.2	mv_K05C4.4	WT	WT
F31D4.8	W02D9.10	mv_F31D4.8	mv_W02D9.10	WT	WT
F31E8.5	Y43F8A.2	mv_F31E8.5	mv_Y43F8A.2	WT	WT
F32A11.1	W09H1.3	mv_F32A11.1	mv_W09H1.3	WT	WT

F32B4.6	R05D7.4	mv_F32B4.6	mv_R05D7.4	WT	WT
F33D11.11	F42G2.5	JA_F33D11.11	mv_F42G2.5	WT	WT
F35C11.2	M05D6.3	mv_F35C11.2	JA_F35C11.2	WT	WT
F35C11.3	M05D6.1	mv_F35C11.3	JA_F35C11.3	WT	WT
F35C5.1	W01B6.8	mv_F35C5.1	mv_W01B6.8	WT	WT
F35H12.2	T24D11.1	mv_F35H12.2	mv_T24D11.1	WT	WT
F36A2.9	T19D7.7	mv_F36A2.9	JA_T19D7.1	WT	WT
F38A5.6	W06D4.3	mv_F38A5.6	mv_W06D4.3	WT	WT
F38E11.7	W03D2.5	mv_F38E11.7	JA_W03D2.5	WT	WT
F39G3.1	M88.1	mv_F39G3.1	mv_M88.1	WT	WT
F40A3.7	F59B2.13	mv_F40A3.7	mv_F59B2.13	WT	WT
F40D4.13	T11F9.10	mv_F40D4.13	mv_T11F9.10	WT	WT
F40G12.9	T19H12.6	mv_F40G12.9	mv_T19H12.6	WT	WT
F41E7.6	T20B3.1	mv_F41E7.6	mv_T20B3.1	WT	WT
F41H10.5	M02B1.3	mv_F41H10.5	mv_M02B1.3	WT	WT
F43C11.3	ZK512.7	mv_F43C11.3	mv_ZK512.7	WT	WT
F43G6.11	Y51H1A.5	JA_F43G6.4	mv_Y51H1A.5	WT	WT
F43G6.5	T15H9.6	mv_F43G6.5	mv_T15H9.6	WT	WT
F43G9.6	T05E8.1	JA_F43G9.6	mv_T05E8.1	WT	WT
F44B9.3	F44B9.4	mv_F44B9.3	mv_F44B9.4	WT	WT
F44F1.1	F44F1.3	mv_F44F1.1	mv_F44F1.3	WT	WT
F45D3.3	F45D3.4	mv_F45D3.3	mv_F45D3.4	WT	WT
F45G2.6	Y110A7A.2	mv_F45G2.6	mv_Y110A7A.2	WT	WT
F46A9.1	F46A9.2	mv_F46A9.1	mv_F46A9.2	WT	WT
F46A9.3	F52E4.4	mv_F46A9.3	JA_F52E4.4	WT	WT
F46B3.11	F46B3.12	mv_F46B3.11	mv_F46B3.12	WT	WT
F46C8.5	K03E6.1	mv_F46C8.5	mv_K03E6.1	WT	WT
F46F11.2	M01E11.5	mv_F46F11.2	JA_M01E11.5	WT	WT
F46F5.1	T04B8.1	mv_F46F5.1	mv_T04B8.1	WT	WT
F46H6.2	F54G8.2	mv_F46H6.2	mv_F54G8.2	WT	WT
F47B10.9	F56C3.9	mv_F47B10.9	mv_F56C3.9	WT	WT
F47B8.3	F47B8.4	mv_F47B8.3	mv_F47B8.4	WT	WT
F47B8.6	F47B8.8	mv_F47B8.6	mv_F47B8.8	WT	WT
F47D12.7	T16H12.6	mv_F47D12.7	mv_T16H12.6	WT	WT
F47G6.4	Y66H1A.6	mv_F47G6.4	mv_Y66H1A.6	WT	WT
F49B2.3	Y116A8B.1	mv_F49B2.3	mv_Y116A8B.1	WT	WT
F49E12.10	F49E12.9	mv_F49E12.10	mv_F49E12.9	WT	WT
F49E2.1	F49H6.5	mv_F49E2.1	mv_F49H6.5	WT	WT
F52D2.9	T26H2.6	mv_F52D2.9	mv_T26H2.6	WT	WT
F52E1.5	F52E1.6	mv_F52E1.5	mv_F52E1.6	WT	WT
F52H2.6	Y7A5A.1	mv_F52H2.6	mv_Y7A5A.1	WT	WT
F52H3.3	Y75B12B.4	mv_F52H3.3	mv_Y75B12B.4	WT	WT
F53C11.7	F53C11.8	mv_F53C11.7	mv_F53C11.8	WT	WT
F53C3.12	Y46G5A.24	JA_F53C3.12	mv_Y46G5A.24	WT	WT
F53C3.13	T28D9.3	mv_F53C3.13	mv_T28D9.3	WT	WT
F53F10.2	K11C4.4	mv_F53F10.2	SA_yk337g11	WT	WT
F54D12.1	F54D12.7	mv_F54D12.1	mv_F54D12.7	WT	WT

F54D5.3	F54D5.4	mv_F54D5.3	mv_F54D5.4	WT	WT
F54F7.1	Y111B2A.16	mv_F54F7.1	JA_Y111B2D.e	WT	WT
F54F7.6	Y105C5B.20	mv_F54F7.6	mv_Y105C5B.20	WT	WT
F54H12.4	Y57G11C.18	mv_F54H12.4	mv_Y57G11C.18	WT	WT
F55B12.6	Y9C9A.2	mv_F55B12.6	mv_Y9C9A.2	WT	WT
F55F3.1	Y47D3A.15	mv_F55F3.1	mv_Y47D3A.15	WT	WT
F56A12.2	M04G7.3	mv_F56A12.2	mv_M04G7.3	WT	WT
F56D5.5	Y38E10A.3	mv_F56D5.5	mv_Y38E10A.3	WT	WT
F56G4.2	F56G4.3	JA_F56G4.2	mv_F56G4.3	WT	WT
F57F4.1	T07D3.9	mv_F57F4.1	JA_T07D3.9	WT	WT
F57F5.2	T07D1.1	mv_F57F5.2	mv_T07D1.1	WT	WT
F58E1.10	F58E1.11	mv_F58E1.10	mv_F58E1.11	WT	WT
F58E2.2	K03D3.2	mv_F58E2.2	mv_K03D3.2	WT	WT
F58E2.3	F58E2.4	mv_F58E2.3	mv_F58E2.4	WT	WT
F58E6.3	W02C12.1	mv_F58E6.3	mv_W02C12.1	WT	WT
F59A6.5	W02B8.2	mv_F59A6.5	mv_W02B8.2	WT	WT
F59B2.8	F59B2.9	mv_F59B2.8	mv_F59B2.9	WT	WT
F59F4.4	T06E8.1	mv_F59F4.4	mv_T06E8.1	WT	WT
H06H21.10	Y49E10.11	mv_H06H21.10	mv_Y49E10.11	WT	WT
H17B01.1	R09B5.11	mv_H17B01.1a	mv_R09B5.11	WT	WT
H25P19.1	T03D3.5	mv_H25P19.1	mv_T03D3.5	WT	WT
H42K12.1	W04B5.5	JA_H42K12.2	mv_W04B5.5	WT	WT
JC8.11	T23B5.1	mv_JC8.11	mv_T23B5.1	WT	WT
K01A2.8	K01A2.9	mv_K01A2.8a	mv_K01A2.9	WT	WT
K01D12.15	K01D12.7	JA_K01D12.15	mv_K01D12.7	WT	WT
K01G5.2	K08H2.6	mv_K01G5.2c	mv_K08H2.6	WT	WT
K02E7.5	M01D1.2	mv_K02E7.5	mv_M01D1.2	WT	WT
K02F3.6	K07C5.5	mv_K02F3.6	mv_K07C5.5	WT	WT
K02G10.5	Y70G10A.3	mv_K02G10.5	mv_Y70G10A.3	WT	WT
K03H6.1	K03H6.5	mv_K03H6.1	mv_K03H6.5	WT	WT
K05D4.6	T09F5.8	mv_K05D4.6	mv_T09F5.8	WT	WT
K05F1.6	ZK455.8	mv_K05F1.6	mv_ZK455.8	WT	WT
K05F1.9	ZK354.7	mv_K05F1.9	mv_ZK354.7	WT	WT
K07A9.2	K11E8.1	JA_K07A9.2	mv_K11E8.1c	WT	WT
K07C11.4	R12A1.4	mv_K07C11.4	JA_R12A1.4	WT	WT
K07E1.1	T01D1.4	mv_K07E1.1	mv_T01D1.4	WT	WT
K07E8.5	T14C1.1	mv_K07E8.5	mv_T14C1.1	WT	WT
K07H8.2	ZK185.2	mv_K07H8.2	mv_ZK185.2	WT	WT
K08H10.7	ZK218.8	mv_K08H10.7	mv_ZK218.8	WT	WT
K09E4.5	ZK622.2	mv_K09E4.5	mv_ZK622.2	WT	WT
K10C9.7	Y39A1A.18	mv_K10C9.7	mv_Y39A1A.18	WT	WT
K10F12.3	R05G6.8	mv_K10F12.3	mv_R05G6.8	WT	WT
K10H10.7	ZK945.8	mv_K10H10.7	mv_ZK945.8	WT	WT
K11G12.3	K11G12.4	mv_K11G12.3	mv_K11G12.4	WT	WT
K11H12.6	K11H12.7	mv_K11H12.6	mv_K11H12.7	WT	WT
M28.2	M28.4	mv_M28.2	mv_M28.4	WT	WT
R02C2.3	Y39H10A.2	mv_R02C2.3	SA_yk260b6	WT	WT

R02C2.5	W03D2.10	mv_R02C2.5	mv_W03D2.10	WT	WT
R03H10.4	R03H10.5	mv_R03H10.4	mv_R03H10.5	WT	WT
R06A4.2	T12E12.3	mv_R06A4.2	mv_T12E12.3	WT	WT
R06C1.4	R09B3.2	mv_R06C1.4	mv_R09B3.2	WT	WT
R06C7.7	Y48G1A.6	mv_R06C7.7	JA_Y48G1A_53.a	WT	WT
R07B7.4	R07B7.5	mv_R07B7.4	JA_R07B7.5	WT	WT
R07C3.14	R07C3.5	mv_R07C3.14	mv_R07C3.5	WT	WT
R08C7.7	Y116A8C.40	mv_R08C7.7	mv_Y116A8C.40	WT	WT
R08F11.6	R09B5.4	mv_R08F11.6	mv_R09B5.4	WT	WT
R08H2.7	T09F5.5	mv_R08H2.7	mv_T09F5.5	WT	WT
R09B5.1	Y38A10A.4	mv_R09B5.1	JA_Y38A10A.4	WT	WT
R09B5.10	R09B5.2	mv_R09B5.10	mv_R09B5.2	WT	WT
R09H10.3	ZK697.8	mv_R09H10.3	mv_ZK697.8	WT	WT
R11A5.4	W05G11.6	mv_R11A5.4	mv_W05G11.6	WT	WT
R11G10.2	Y37A1B.5	mv_R11G10.2	mv_Y37A1B.5	WT	WT
R13A5.10	Y48A6B.7	mv_R13A5.10	mv_Y48A6B.7	WT	WT
R57.2	Y65B4BL.1	mv_R57.2	mv_Y65B4BL.1	WT	WT
R74.5	T07D1.4	mv_R74.5	JA_T07D1.4	WT	WT
T01A4.1	T01A4.2	mv_T01A4.1	mv_T01A4.2	WT	WT
T01B10.1	W05E7.1	mv_T01B10.1	mv_W05E7.1	WT	WT
T01B10.2	W05E7.3	mv_T01B10.2	JA_W05E7.1	WT	WT
T02G5.11	ZK1127.1	mv_T02G5.11	mv_ZK1127.1	WT	WT
T04D3.1	ZK1053.4	mv_T04D3.1	mv_ZK1053.4	WT	WT
T04H1.2	Y38C9A.2	JA_T04H1.2	JA_Y38C9A.2	WT	WT
T04H1.7	T04H1.8	mv_T04H1.7	mv_T04H1.8	WT	WT
T05H4.13	T08B1.3	mv_T05H4.13	mv_T08B1.3	WT	WT
T07C4.5	Y5F2A.2	mv_T07C4.5	mv_Y5F2A.2	WT	WT
T07H3.1	T07H3.2	mv_T07H3.1	mv_T07H3.2	WT	WT
T08D2.1	Y60A3A.9	mv_T08D2.1	mv_Y60A3A.9	WT	WT
T13F3.2	T13F3.3	mv_T13F3.2	mv_T13F3.3	WT	WT
T14G11.3	W06H3.1	mv_T14G11.3	mv_W06H3.1	WT	WT
T14G8.3	T24H7.2	mv_T14G8.3	mv_T24H7.2	WT	WT
T16H12.8	Y73C8C.9	mv_T16H12.8	mv_Y73C8C.9	WT	WT
T17A3.8	Y32B12A.1	mv_T17A3.8	mv_Y32B12A.1	WT	WT
T17A3.9	VW02B12L.4	mv_T17A3.9	JA_VW02B12L.4	WT	WT
T18D3.3	Y39E4A.2	mv_T18D3.3	mv_Y39E4A.2a	WT	WT
T19H12.4	T19H12.5	mv_T19H12.4	mv_T19H12.5	WT	WT
T20F7.1	Y48G9A.11	mv_T20F7.1	JA_Y67D8A_382.c	WT	WT
T21C9.11	T21C9.13	mv_T21C9.11	mv_T21C9.13	WT	WT
T23C6.1	Y71F9B.10	mv_T23C6.1	mv_Y71F9B.10	WT	WT
T23D5.10	T23D5.12	mv_T23D5.10	mv_T23D5.12	WT	WT
T23G11.7	Y34B4A.2	mv_T23G11.7	JA_Y34B4A.f	WT	WT
T24H7.5	W09D10.2	mv_T24H7.5a	mv_W09D10.2	WT	WT
T26C11.6	T26C11.7	mv_T26C11.6	mv_T26C11.7	WT	WT
T27A1.5	Y43F4B.7	mv_T27A1.5	mv_Y43F4B.7	WT	WT
T27C10.1	T27C10.2	mv_T27C10.1	mv_T27C10.2	WT	WT
T28H11.2	T28H11.3	mv_T28H11.2	mv_T28H11.3	WT	WT

W01C8.6	ZC416.8	mv_W01C8.6	JA_ZC416.8	WT	WT
W02D9.4	ZK154.5	mv_W02D9.4	mv_ZK154.5	WT	WT
W03G9.2	Y45G5AM.2	mv_W03G9.2	SA_yk355b1	WT	WT
W06D4.6	Y116A8C.13	mv_W06D4.6	mv_Y116A8C.13	WT	WT
W09D6.4	Y47D3A.13	mv_W09D6.4	mv_Y47D3A.13	WT	WT
W09H1.5	Y48A6B.9	mv_W09H1.5	mv_Y48A6B.9	WT	WT
Y116A8A.2	Y116A8A.7	mv_Y116A8A.2	mv_Y116A8A.7	WT	WT
Y116A8A.4	Y116A8A.6	mv_Y116A8A.4	mv_Y116A8A.6	WT	WT
Y116A8C.11	Y116A8C.18	mv_Y116A8C.11	mv_Y116A8C.18	WT	WT
Y116A8C.14	Y116A8C.16	mv_Y116A8C.14	mv_Y116A8C.16	WT	WT
Y11D7A.1	ZK1320.9	mv_Y11D7A.1	mv_ZK1320.9	WT	WT
Y17D7C.2	Y69H2.9	mv_Y17D7C.2	mv_Y69H2.9	WT	WT
Y17G7B.10	Y77E11A.2	mv_Y17G7B.10a	mv_Y77E11A.2	WT	WT
Y17G9B.1	Y75B8A.25	mv_Y17G9B.1	mv_Y75B8A.25	WT	WT
Y24D9B.1	Y51H4A.13	SA_yk307h6	mv_Y51H4A.13	WT	WT
Y32B12C.3	Y70C5A.2	mv_Y32B12C.3	mv_Y70C5A.2	WT	WT
Y38C1AB.4	Y38C1AB.8	mv_Y38C1AB.4	mv_Y38C1AB.8	WT	WT
Y38H6C.16	ZK84.2	mv_Y38H6C.16	mv_ZK84.2	WT	WT
Y39E4A.3	Y43F4A.4	JA_Y39E4A.3	mv_Y43F4A.4	WT	WT
Y43F11A.6	Y53C10A.1	mv_Y43F11A.6	mv_Y53C10A.1	WT	WT
Y47D3A.1	Y48A6C.5	mv_Y47D3A.1	JA_Y48A6C.5	WT	WT
Y47G6A.1	Y47G6A.2	mv_Y47G6A.1	mv_Y47G6A.2	WT	WT
Y47H9C.10	Y47H9C.11	mv_Y47H9C.10	mv_Y47H9C.11	WT	WT
Y47H9C.4	Y64G10A.7	mv_Y47H9C.4	mv_Y64G10A.7	WT	WT
Y53F4B.36	ZC317.6	mv_Y53F4B.36	mv_ZC317.6	WT	WT
Y55B1AR.3	Y55B1AR.4	mv_Y55B1AR.3	mv_Y55B1AR.4	WT	WT
Y57G11C.5	Y73F8A.20	mv_Y57G11C.5	mv_Y73F8A.20	WT	WT
Y75B7B.2	ZK1005.1	mv_Y75B7B.2	mv_ZK1005.1	WT	WT
Y75D11A.2	Y75D11A.3	mv_Y75D11A.2	mv_Y75D11A.3	WT	WT
Y82E9BL.10	Y82E9BL.11	JA_Y119D3_457.b	JA_Y119D3_457.c	WT	WT
Y82E9BR.1	Y82E9BR.17	JA_Y119D3_448.a	mv_Y82E9BR.17	WT	WT
ZC132.9	ZK218.4	mv_ZC132.9	mv_ZK218.4	WT	WT
ZC412.3	ZC412.4	mv_ZC412.3	mv_ZC412.4	WT	WT
ZC412.5	ZK512.8	mv_ZC412.5	mv_ZK512.8	WT	WT
